# MRI changes of pelvic floor and pubic bone observed in primiparous women after childbirth by normal vaginal delivery

**DOI:** 10.1007/s00404-016-4023-z

**Published:** 2016-02-10

**Authors:** Minghai Shi, Shiyao Shang, Bing Xie, Jianliu Wang, Bin Hu, Xueying Sun, Jin Wu, Nan Hong

**Affiliations:** Department of Radiology, Peking University People’s Hospital, Beijing, 100044 China; Department of Radiology, Guangdong General Hospital, Guangzhou, 510080 China; Department of Gynaecology and Obstetrics, Peking University People’s Hospital, Beijing, 100044 China; Department of Ultrosonography, A la Shan Central Hospital, A la Shan, 750306 Inner Mongolia China; Department of Radiology, Peking University People’s Hospital, No. 11 Xizhimen South Street, Xicheng District, Beijing, 100044 China

**Keywords:** Levator ani tears, Pubic bone edema, Fracture, Magnetic resonance imaging

## Abstract

**Objective:**

To identify and characterize changes in the pelvic floor and pubic bone, using magnetic resonance imaging, in primiparous women with normal vaginal delivery, in comparison with nulliparous women.

**Methods:**

Pelvic MR images from ten primiparous women, 6–7 weeks after normal vaginal delivery, and ten nulliparous women were obtained from January to April 2014. The selected women were scanned using a multiplanar T2FRFSE sequence and T2fsFRFSE sequence. Changes in the pelvic floor and pubic bone in primiparous women, including tears of the levator ani fibers, pubic bone edema, and fractures, were assessed on the MR images in comparison with images from normal nulliparous women. Injury to the levator ani was evaluated and scored. The incidence, location and the extent of injury to the levator ani and pubic bone were characterized further.

**Results:**

Eight out of ten primiparous women had imaging abnormalities after normal vaginal delivery. Three women had unilateral tears of the pubococcygeus, in which the defect in the muscle was located at or near its origin at the pubic bone, and one had a pubococcygeus tear accompanied by bilateral spilling of the vagina. Four women had partial tears of the iliococcygeus: one was a bilateral tear, and three were unilateral. None had a tear of the coccygeus. Eight women had pubic bone marrow edema; one was accompanied by a pubic bone fracture line. None of the nulliparous women had any abnormality of the pelvic floor and pubic bone.

**Conclusion:**

Abnormalities of the pelvic floor and pubic bone were observed in primiparous women but not in nulliparous women. In primiparous women, most levator ani muscle tears are at or near the point of origin, and pubococcygeus injuries are usually accompanied by pubic bone marrow edema.

## Introduction

The levator hiatus is the largest potential hernia portal within the envelope of the abdominal cavity [1]; it is enclosed by the pelvic floor, which acts as a support for the pelvic and abdominal organs. The levator ani muscle is thought to be of central importance for pelvic organ support [2]. One of the main functions of the pelvic floor is to maintain urinary and fecal continence [3]. Pelvic organ prolapse, urinary incontinence and fecal incontinence are the most common disorders of the pelvic floor [3]. Pelvic floor dysfunction is common in women, and can significantly affect daily activities and quality of life. In a recent study, the lifetime risk of surgery for either stress incontinence or pelvic organ prolapse was 20.0 % [4].

It is well established that the levator ani muscle plays an important role in maintaining the function of the pelvic floor [5–7]. Pelvic floor disorders, including urinary and fecal incontinence and pelvic organ prolapse, are more prevalent among women who have delivered at least one child [8–10], and vaginal parity is the most consistently associated risk factor [11]. Levator ani muscle avulsion can occur during vaginal delivery; in particular, avulsions of the pubococcygeus appear to be clinically relevant as an independent risk factor for pelvic organ prolapse [12].

Each part of the levator ani muscle differs in its origin and insertion points and therefore has a unique function. Injury to each individual part results in a unique dysfunction. Visible defects of the levator ani muscle have been observed in magnetic resonance (MR) images and three-dimensional (3D) ultrasound of women after vaginal delivery [13, 14]. It has been reported that visible defects in the pubovisceral portion of the levator ani muscles occur in 20–36 % of primiparous women after vaginal delivery [13, 15], in contrast to nulliparous women in whom such abnormalities were not found [14, 16, 17]. Injury to the levator ani muscles as a result of vaginal birth has been documented [13].

Accurate assessment of pelvic floor injury through MR imaging can provide help in the diagnosis of pelvic floor disorders and can provide detailed structural evidence. The goal of this study was to identify the damaged portion and the extent of pelvic floor and pubic bone injury in primiparous women after normal vaginal delivery, and to compare them with nulliparous women. Visualizing muscular injuries and bone abnormalities in the pelvic floor by MR imaging requires high spatial and contrast resolution. In our study we used a 3.0T MR scanner which provides images of high spatial and contrast resolution; 3.0T MR imaging can demonstrate the location and the extent of pelvic floor injury and pubic bone injury.

Ten primiparous women underwent MR scanning, 6–7 weeks after normal vaginal delivery, for assessment of pelvic floor and bone abnormalities, and the findings were compared with those from nulliparous women.

## Materials and methods

This study was a single institution study, conducted by investigators at Peking University People’s Hospital, and was approved by the institutional board review. All participants provided written informed consent. Our study groups comprised ten primiparous women following spontaneous vaginal delivery and ten nulliparous women matched on age and body mass index (BMI). Our exclusion criteria included maternal age younger than 15 or older than 50 years, delivery at less than 37 weeks’ gestation, placenta previa, known fetal congenital anomaly, stillbirth, prior myomectomy or abruption. The age range of the primiparous group was 26–34 years with a mean age of 28.5 years, and the BMI of this group was 20.2–27.3 with a mean of 20.5 kg/m^2^. The age range of the nulliparous group was from 23 to 29 years with a mean age of 26.2 years, and the BMI of this group ranged from 17.5 to 22.0 with a mean of 19.7 kg/m^2^.

The women in both the primiparous and nulliparous groups completed the same MR scan sequence. The primiparous women were all scanned about 6–7 weeks after delivery. The nulliparous women were scanned during a routine visit for other medical conditions. The MR scan was performed after participants had emptied their bladder, with the patient in the supine position at rest. The MR images of the primiparous group and nulliparous group were studied and compared at the same anatomical level. The MR imaging (750 3.0 Tesla, Signa; General Electric Medical Systems, Milwaukee, WI; 32 channel torso coil positioned over the pelvis) included a coronal, axial, and sagittal T2 fast recovery fast spin echo (FRFSE) sequence (TR/TE = 3000/102 ms, field-of-view = 28 cm; 320 × 320 matrix, number of signal averages 2; and slice thickness/gap = 4 mm/1 mm), and a T2 fat saturation fast recovery fast spin echo (fsFRFSE) sequence. The MRI images were reviewed and assessed independently by two radiologists using the standard monitor of the picture archiving and communication system (PACS); consensus was achieved by reviewing the images together. The pelvic floor and pubic bone were assessed while blinded to clinical risk factors. Tears of the levator ani muscle, edema, and fracture of the pubic bone were assessed in all MR images. Each side of an injury to the levator ani was assessed and scored separately. A tear of the levator ani was assessed by recording the absence of muscle fibers in at least one 4 mm section in the axial, sagittal and/or coronal planes. Each tear was scored from 0 to 3 as follows: score 0, normal; score 1, less than 50 % of muscle fibers missing; score 2, more than 50 % of muscle fibers missing; score 3, complete loss of muscle mass. Increased T2fsFRFSE signal of the pubic bone on MR images, the presence and location of any fracture line, the location and type of injury, and the extent of avulsion were recorded. The clinical information on the primiparous and nulliparous groups was collected by review of medical records.

## Results

There were ten primiparous women with normal vaginal delivery and ten nulliparous women in the study group; the groups were matched on age and BMI. All the women were seen in the radiology department in Peking University People’s Hospital between January and April 2014. Clinically, none of the women presented with pelvic organ prolapse or stress urinary or fecal incontinence, and gynecological examination did not reveal any obvious abnormality. Among the primiparous group, none had experienced anal sphincter laceration, a prolonged second stage of labor, precipitous delivery or infant shoulder dystocia during delivery.

**Fig. 1:**
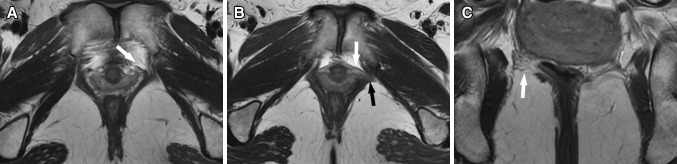
T2-weighted MR images of pubococcygeus and iliococcygeus tears. In **a**, an axial image from a 26-year-old primiparous woman shows partial (<50 %) left pubococcygeus avulsion near the pubic bone (*white arrow*); the right pubococcygeus muscle bulk is normal. In **b**, an axial image from a 31-year-old primiparous woman shows partial (<50 %) left pubococcygeus avulsion near the pubic bone (*black arrow*) with bilateral spilling of the vagina; the vaginal spilling on the left side is more severe (*white arrow*). In **c**, a coronal image from a 26-year-old primiparous woman shows partial right iliococcygeus tears near the obturator internus fascia (*white arrow*)

**Fig. 2:**
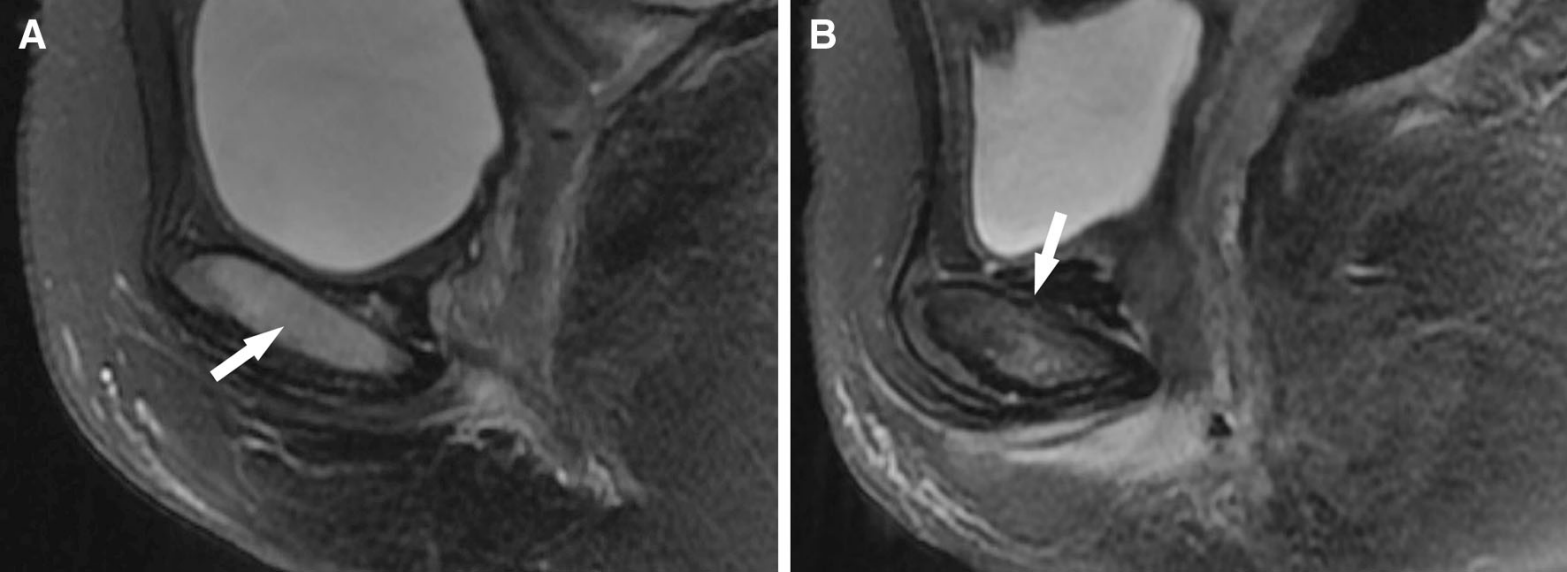
T2fsFRFSE images of pubic bone edema and cortex microfracture. In **a**, a saggital image from a 24-year-old primiparous woman shows pubic bone marrow edema (*white arrow*). In **b**, a saggital image from a 31-year-old primiparous woman shows pubic bone marrow edema and fracture (*white arrow* at the superior symphysis)

Pubococcygeus injury was found in three out of the ten primiparous women: three women had a score of 1 on one side (Fig. 1a); seven patients demonstrated no pubococcygeus tear. One unilateral pubococcygeus injury was accompanied by bilateral spilling of the vagina, and the spilling of the vagina on the side with the pubococcygeus tear was more serious (Fig. 1b). Local injury to the iliococcygeus was seen in four primiparous women: one of them had bilateral local tears, and three had a unilateral local tear (Fig. 1c). Injury to the coccygeus was not seen in any of the primiparous women. However, injury to the pubic bone was seen in eight primiparous women; among them five had bilateral bone marrow edema and three women had unilateral edema (Fig. 2a). One case of edema was accompanied by a subtle fracture of the ipsilateral pubic bone (Fig. 2b).

The majority of injuries to the levator ani were located at the point of origin, and tears of the pubococcygeus were located at the pubic origin and in the anterior portion. The tears of the iliococcygeus were located at or near the fascia of the obturator internus. All tears of the pubococcygeus were associated with focal pubic bone marrow edema, but some cases of pubic bone edema were not accompanied by pubococcygeus tears, and only one instance of pubic bone edema was accompanied by a local fracture. These injuries to the pelvic floor and pubic bone of primiparous women occurred singly or in combination.

Muscle tears or obvious abnormality of the pelvic floor and pubic bone were not found on MR imaging of any nulliparous woman.

## Discussion

Although none of the primiparous women in our study group experienced any adverse events during normal vaginal delivery, mild injuries to the levator ani and pubic bone were demonstrated on MR images obtained 6–7 weeks postpartum. We chose this imaging time to minimize the influence of soft tissue edema caused by vaginal delivery [18, 19]. In strong contrast, similar injuries were not found in any of the nulliparous women. Therefore even uneventful natural labor can lead to mild injury to the levator ani and pubic bone.

In our study, all levator ani injuries were tears in the pubococcygeus and/or iliococcygeus; obvious injury to the coccygeus was not observed. In comparison with the pubococcygeus and iliococcygeus, there is a greater distance between the coccygeus and the levator hiatus, which may explain the absence of injury to the coccygeus during vaginal birth. Tears of the pubococcygeus are usually associated with bone marrow edema in the pubic bone and are occasionally associated with local fracture. However, we also observed bone marrow edema in the pubic bone without evidence of tears in the pubococcygeus, which suggests that pubic bone edema occurs more frequently than pubococcygeus tears. In addition, the extent of pubic bone marrow edema and local fracture was correlated with the extent and side of the pubococcygeus injury.

The injuries observed in the pubic portion of the pubococcygeus and the pubic bone support the hypothesis that tears of the pubic portion of the levator ani fibers are a triggering mechanism for pelvic floor injury during vaginal delivery. Based on our observations, the medial pubic part of the pubococcygeus is more vulnerable to injury, because we did not observe any tears in the lateral part of the pubococcygeus origin. One pubococcygeus tear was accompanied by bilateral spilling of the vagina; the extent of the spilling on the side of the muscle tear was greater than that on the contralateral side, suggesting that lateral vaginal spilling may be associated with the side and extent of pubococcygeus tears.

While MR-detectable changes in the pubic bone occurred in all groups in a previous study, they were more frequently associated with labor and with delivery-related risk factors for injury to the pelvic floor muscles [19]. Bone marrow edema, also named “bone marrow contusion”, is an indirect sign of injury to the pubic bone portion of the pubococcygeus, and indicates non-specific stress injury within the bone. In this study, eight out of ten primiparous women had pubic bone marrow edema. Bone marrow edema is common following all types of delivery, and pubic bone fractures occur when the stress exceeds trabecular or cortical strength. In this study, one pubic bone microfracture was noted in the primiparous group, and this woman also had an obvious ipsilateral pubococcygeus tear. This indicates that pubic bone fracture may be related to the extent and side of pubococcygeus injury. In addition, tears in the iliococcygeus tears were relatively common findings in the primiparous women after normal vaginal delivery.

The location of injuries to the levator ani in our study supports the hypothesis that the origin point of the levator ani is injured more often than other parts and the insertion points; in addition, the medial parts of the pubococcygeus were injured more often than the lateral part. Muscles in general are most vulnerable to tears at the myotendinous junction [20, 21], and most levator ani injuries in our study were located at this part. This pattern of muscle tearing occurred in the same region as the muscle defects seen in women with pelvic organ prolapse [22].

Symptoms are only weakly correlated with the extent of prolapse [23, 24]. In this study, the injuries to the pelvic floor and pubic bone were mild, and the nulliparous women did not present with any symptoms of pelvic organ prolapse, or stress urinary or fecal incontinence, and gynecological examination was normal. It is likely that the time we selected for follow-up of postpartum symptoms was too early, and that the extent of injury to the pelvic floor and pubic bone that is demonstrated by highly sensitive imaging techniques does not cause symptoms in primiparous women. Women with signs of prolapse are more likely to be older (10 or more years postmenopausal), obese, and to have had at least one vaginal birth [25]. However, when compared with the nulliparous group, the MR imaging of primiparous women after normal vaginal delivery demonstrated obvious injuries to the levator ani and pubic bone which strongly support the contention that vaginal delivery is a major risk factor for pelvic floor injury.

This study was based in a single institution and the sample size was relatively small, yet it laid the ground for large cohort study. A multi-institutional study with large sample size is warranted to characterize fully the site of levator ani tears, and edema and fracture of the pubic bone, to determine the significance of these results and the association with other clinical findings. In summary, our study supports the hypothesis that the primary mechanism of pelvic floor injury associated with vaginal delivery is likely to be due to levator ani muscle tears, most commonly located at the point of origin.

## References

[1] Dietz HP, Shek C, De Leon J, Steensma AB (2008). Ballooning of the levator hiatus. Ultrasound Obstet Gynecol.

[2] DeLancey JO (2005). The hidden epidemic of pelvic floor dysfunction: achievable goals for improved prevention and treatment. Am J Obstet Gynecol.

[3] Lammers K, Prokop M, Vierhout ME, Kluivers KB, Futterer JJ (2013). A pictorial overview of pubovisceral muscle avulsions on pelvic floor magnetic resonance imaging. Insights Imaging.

[4] Wu JM, Matthews CA, Conover MM, Pate V, Jonsson Funk M (2014). Lifetime risk of stress urinary incontinence or pelvic organ prolapse surgery. Obstet Gynecol.

[5] Hoyte L, Jakab M, Warfield SK, Shott S, Flesh G, Fielding JR (2004). Levator ani thickness variations in symptomatic and asymptomatic women using magnetic resonance-based 3-dimensional color mapping. Am J Obstet Gynecol.

[6] Ashton-Miller JA, Delancey JO (2009). On the biomechanics of vaginal birth and common sequelae. Annu Rev Biomed Eng.

[7] Morgan DM, Kaur G, Hsu Y, Fenner DE, Guire K, Miller J, Ashton-Miller JA, Delancey JO (2005). Does vaginal closure force differ in the supine and standing positions?. Am J Obstet Gynecol.

[8] Nygaard I, Barber MD, Burgio KL, Kenton K, Meikle S, Schaffer J, Spino C, Whitehead WE, Wu J, Brody DJ, Pelvic Floor Disorders Network (2008). Prevalence of symptomatic pelvic floor disorders in US women. JAMA.

[9] Rortveit G, Daltveit AK, Hannestad YS, Hunskaar S, Norwegian ES (2003). Urinary incontinence after vaginal delivery or cesarean section. N Engl J Med.

[10] Mant J, Painter R, Vessey M (1997). Epidemiology of genital prolapse: observations from the Oxford Family Planning Association Study. Br J Obstet Gynaecol.

[11] Hendrix SL, Clark A, Nygaard I, Aragaki A, Barnabei V, McTiernan A (2002). Pelvic organ prolapse in the Women’s Health Initiative: gravity and gravidity. Am J Obstet Gynecol.

[12] Lammers K, Futterer JJ, Prokop M, Vierhout ME, Kluivers KB (2012). Diagnosing pubovisceral avulsions: a systematic review of the clinical relevance of a prevalent anatomical defect. Int Urogynecol J.

[13] DeLancey JO, Kearney R, Chou Q, Speights S, Binno S (2003). The appearance of levator ani muscle abnormalities in magnetic resonance images after vaginal delivery. Obstet Gynecol.

[14] Ying T, Li Q, Xu L, Liu F, Hu B (2012). Three-dimensional ultrasound appearance of pelvic floor in nulliparous women and pelvic organ prolapse women. Int J Med Sci.

[15] Dietz HP, Lanzarone V (2005). Levator trauma after vaginal delivery. Obstet Gynecol.

[16] Margulies RU, Hsu Y, Kearney R, Stein T, Umek WH, DeLancey JO (2006). Appearance of the levator ani muscle subdivisions in magnetic resonance images. Obstet Gynecol.

[17] Hoyte L, Damaser MS (2007). Magnetic resonance-based female pelvic anatomy as relevant for maternal childbirth injury simulations. Ann N Y Acad Sci.

[18] Tunn R, DeLancey JO, Howard D, Thorp JM, Ashton-Miller JA, Quint LE (1999). MR imaging of levator ani muscle recovery following vaginal delivery. Int Urogynecol J Pelvic Floor Dysfunct.

[19] Brandon C, Jacobson JA, Low LK, Park L, DeLancey J, Miller J (2012). Pubic bone injuries in primiparous women: magnetic resonance imaging in detection and differential diagnosis of structural injury. Ultrasound Obstet Gynecol.

[20] Benjamin M, Toumi H, Ralphs JR, Bydder G, Best TM, Milz S (2006). Where tendons and ligaments meet bone: attachment sites (‘entheses’) in relation to exercise and/or mechanical load. J Anat.

[21] Connell DA, Schneider-Kolsky ME, Hoving JL, Malara F, Buchbinder R, Koulouris G, Burke F, Bass C (2004). Longitudinal study comparing sonographic and MRI assessments of acute and healing hamstring injuries. AJR Am J Roentgenol.

[22] DeLancey JO, Morgan DM, Fenner DE, Kearney R, Guire K, Miller JM, Hussain H, Umek W, Hsu Y, Ashton-Miller JA (2007). Comparison of levator ani muscle defects and function in women with and without pelvic organ prolapse. Obstet Gynecol.

[23] Gutman RE, Ford DE, Quiroz LH, Shippey SH, Handa VL (2008). Is there a pelvic organ prolapse threshold that predicts pelvic floor symptoms?. Am J Obstet Gynecol.

[24] Bradley CS, Nygaard IE (2005). Vaginal wall descensus and pelvic floor symptoms in older women. Obstet Gynecol.

[25] Mothes AR, Radosa MP, Altendorf-Hofmann A, Runnebaum IB (2015). Risk index for pelvic organ prolapse based on established individual risk factors. Arch Gynecol Obstet.

